# Influence of microRNA on the Maintenance of Human Iron Metabolism

**DOI:** 10.3390/nu5072611

**Published:** 2013-07-10

**Authors:** McKale Davis, Stephen Clarke

**Affiliations:** Department of Nutritional Sciences, Oklahoma State University, Stillwater, OK 74078, USA; E-Mail: mckale.davis@okstate.edu

**Keywords:** diet, cell metabolism, iron homeostasis, microRNA regulation

## Abstract

Iron is an essential nutrient critical for many cellular functions including DNA synthesis, ATP generation, and cellular proliferation. Though essential, excessive iron may contribute to the generation of free radicals capable of damaging cellular lipids, proteins, and nucleic acids. As such, the maintenance and control of cellular iron homeostasis is critical to prevent either iron deficiency or iron toxicity conditions. The maintenance of cellular iron homeostasis is largely coordinated by a family of cytosolic RNA binding proteins known as Iron Regulatory Proteins (IRP) that function to post-transcriptionally control the translation and/or stability of mRNA encoding proteins required for iron uptake, storage, transport, and utilization. More recently, a class of small non-coding RNA known as microRNA (miRNA) has also been implicated in the control of iron metabolism. To date, miRNA have been demonstrated to post-transcriptionally regulate the expression of genes associated with iron acquisition (transferrin receptor and divalent metal transporter), iron export (ferroportin), iron storage (ferritin), iron utilization (ISCU), and coordination of systemic iron homeostasis (HFE and hemojevelin). Given the diversity of miRNA and number of potential mRNA targets, characterizing factors that contribute to alterations in miRNA expression, biogenesis, and processing will enhance our understanding of mechanisms by which cells respond to changes in iron demand and/or iron availability to control cellular iron homeostasis.

## 1. Introduction

Iron, an essential nutrient, is one of the most abundant minerals in the earth’s crust, yet iron deficiency (ID) remains the most common micronutrient deficiency in the world, affecting some 1.6 billion people or nearly 25% of the earth’s population [1]. ID may arise from insufficient absorption of dietary iron, insufficient dietary iron intake, or from complications associated with intestinal parasitic infection. Regardless of the etiology, ID progresses in stages beginning with a depletion of iron stores, followed by diminished erythropoiesis, and finally a reduction in hemoglobin production resulting in anemia [2]. Symptoms of ID include weakness, fatigue, reduced cognitive function in children, and an impaired immune response.

Iron is critical for oxygen transport, DNA synthesis, ATP generation, and cellular proliferation. At the molecular level, ID elicits a cascade of cellular events aimed at conserving iron for the maintenance of these life-preserving functions, though tissue-specific responses and physiologic adaptations to ID are not yet fully understood. Small regulatory RNA molecules called microRNA (miRNA) have an important role in the regulation of many cellular regulatory and disease processes. Current evidence suggests that miRNA may also be key regulators in many facets of human iron homeostasis. This evidence for the role of miRNA in modulating iron homeostasis is underscored by the fact that miRNA processing is, at least in part, a heme-dependent process [3,4]. Thus, determining the roles of miRNA in coordinating the molecular response to changes in iron status may provide fundamental insights into the understanding of how iron homeostasis is maintained, and how alterations in iron sensing can lead to the development of disease.

## 2. microRNA Nomenclature

The first miRNA, lin-4, was discovered in 1993, and was identified as being critically important for developmental timing in *Caenorhabditis* *elegans*, though at the it time was largely considered an anomaly in worm genetics [5]. The next miRNA discovered, let-7, was not identified until 2000 [6]. Intriguingly, the miRNA let-7, also discovered in *C. elegans*, was found to be highly conserved among all animals [7]. Shortly thereafter, in 2001, several additional miRNA were discovered in *Drosophila melanogaster* and in the human HeLa cell line [8,9]. To date (April 2013), 21264 precursor miRNA expressing 25141 mature miRNA have been annotated in 193 species and logged in the latest (release 19) miRBase database repository [10]. Of these, there are currently 1600 annotated human precursor miRNA expressing 2042 mature miRNA. The distinction between precursor and mature miRNA is discussed below.

With thousands of miRNA in numerous species being identified in a relatively short period of time, it was essential to establish criteria to be used in annotating each newly discovered miRNA [11,12]. In addition to creating uniform standards for naming miRNA across species, the nomenclature is also designed to convey at least some minimal biological meaning or context. Each experimentally validated novel miRNA is designated with a unique name following these rules prior to publication, with exceptions being made for lin-4 and let-7 whose names have been retained for historical reasons.

First, miRNA are labeled numerically, and in sequential order with the prefix “mir” followed by a dash, with an un-capitalized “mir-” generally referring to the precursor miRNA, while a capitalized “miR-” generally denotes the mature form. For instance, if the last annotated human precursor miRNA was mir-6724, the next novel published miRNA precursor will be numbered mir-6725. For further clarification, the names are also preceded by 3 letters signifying the species of origin, such as “hsa-” for *Homo sapiens*, “mmu-” for *Mus musculus*, or “dme-” *Drosophila melanogaster*.

Additionally, miRNA with nearly identical structure and sequencing, barring one or two nucleotides, are annotated with a lower case letter such that relationships among miRNA can be inferred (e.g., miR-181a is closely related to miR-181b) [12]. Numbered suffixes, however, designate distinct precursor sequences and genomic loci that express 100% identical mature miRNA [12]. For example, the designation of hsa-mir-6725-1 and hsa-mir-6725-2 would indicate that while these two precursor miRNA may be located in different regions of the genome, both are processed into identical mature miRNA hsa-miR-6725. miRNA which originate from the same precursor are often referred to as a miRNA:miRNA* (or miRNA-star) duplex [13]. With this star or non-star nomenclature, the non-star strand of the duplex represents the predominant functional “guide” strand, and the star strand represents the less abundant and more rapidly turning over “passenger” strand. However, when available sequencing data is not sufficient to designate the predominant strand, a naming convention that identifies the miRNA strand location on the 5′- or 3′-arm of the precursor miRNA is used (e.g., hsa-miR-6725-5p and hsa-miR-6725-3p) [13].

## 3. Iron and Heme in microRNA Processing

In mammals, RNA polymerase II transcribes primary miRNA (pri-miRNA) transcripts that contain at least one hairpin structure consisting of a double-stranded stem and a terminal loop, and may be several kilobases in length [14]. In the nucleus, this pri-miRNA transcript is cleaved at the stem of the hairpin structure by the microprocessor core complex composed of the RNase II-type protein Drosha and its cofactor protein known as DiGeorge syndrome critical region gene 8 (DGCR8) [14,15]. The product of this processing is an ~70 nucleotide long precursor miRNA (pre-miRNA) that is then exported out of the nucleus into the cytoplasm by the nuclear export factor exportin 5 (Exp5) through the recognition of a short 3′-overhang [15]. Upon entry into the cytoplasm, the RNase III-like enzyme Dicer catalyzes the second processing step of “dicing” the pre-miRNA to produce a ~22 nucleotide long miRNA duplex [15,16].

**Figure 1:**
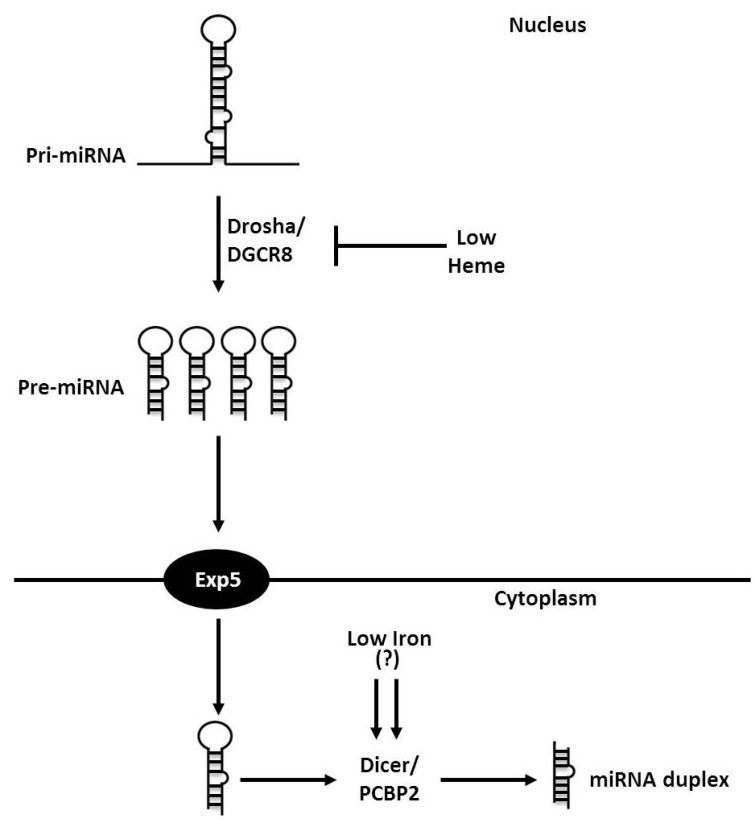
Relationship between cellular iron and miRNA processing. Following their transcription, primary miRNA (pri-miRNA) are cleaved at the stem of the hairpin structure by the RNase II-type protein Drosha and its cofactor DGCR8, a heme-binding protein. Heme-free DGCR8 is less active than heme-bound DGCR8 suggesting that cellular iron status may affect the rate and efficiency of pri-miRNA processing. The product of Drosha/DGCR8 processing is an ~70 nucleotide long precursor miRNA (pre-miRNA) that is exported out of the nucleus into the cytoplasm by the nuclear export factor exportin 5 (Exp5) through the recognition of a short 3′-overhang on the pre-miRNA. Upon entry into the cytoplasm, the RNase III-like enzyme Dicer catalyzes the second processing step of “dicing” the pre-miRNA to produce an ~22 nucleotide long miRNA duplex. Preliminary evidence suggests that iron also regulates the processing of pre-miRNA via the iron-dependent regulation of Dicer activity through its association with poly(rC)-binding protein 2 (Pcbp2), wherein the removal of cytosolic iron, but not heme-iron, enhances pre-miRNA processing. Following cleavage by Dicer, the miRNA duplex is available to be assembled into the RISC to participate in RNA silencing of target mRNA.

Following cleavage by Dicer, the miRNA duplex is loaded onto an Argonaute (Ago) protein. Ago proteins are highly specialized small-RNA-binding proteins that are critical components of RNA-silencing pathways [17]. Following loading onto an Ago protein, one of the two strands (generally referred to as the guide strand) is assembled into the RNA-induced silencing complex (RISC) to facilitate RNA silencing [18]. The loaded RISC is competent to interact with recognition sites known as seed sequences, typically located in the 3′-untranslated region (UTR) of target mRNA, though examples also exist where the seed sequence is located in the 5′UTR or even within the open-reading frame of target mRNA [19,20]. Upon binding to a target sequence, the RISC functions to silence the target mRNA via mRNA degradation or translational repression [14,16]. The opposite, or unloaded strand, often referred to as the passenger or miRNA* strand, was initially thought to be removed from the RISC and degraded, but recent work indicates that these so-called miRNA* strands also have important functional regulatory roles [21,22].

Interestingly and in relation to this review, iron appears to play a critical role in miRNA processing via its physiological role as the functional component in heme. This potential role for iron to participate in miRNA biogenesis was first demonstrated when DGCR8 was identified as a heme-binding protein [4]. Additional studies demonstrated that heme-free DGCR8 was less active than heme-bound DGCR8 and suggests that an impaired ability to synthesize heme as a result of inadequate iron could decrease pri-miRNA processing [4]. In addition to heme availability, the oxidation state of iron in heme affects heme-mediated regulation of DGCR8 [3]. The reduction of ferric heme to ferrous heme abolishes DGCR8 pri-miRNA processing activity thereby affecting the rate and efficiency of pri-miRNA processing [3]. Recent work has now provided evidence that iron also regulates the processing of pre-miRNA via the iron-dependent regulation of Dicer activity through its association with poly(rC)-binding protein 2 (Pcbp2) [23]. Pcbp2 association with Dicer appears to promote the cytosolic processing of pre-miRNA precursors [23]. The effect of Pcbp2 on pre-miRNA processing was enhanced with the removal of cytosolic iron, but not heme-iron, via the use of iron chelators [23]. Figure 1 illustrates key aspects of miRNA processing that may be influenced by iron availability.

## 4. The Pathologic and Physiologic Roles of miRNA in Humans

Soon after the discovery of the first miRNA in humans, the initial studies linking miRNA to human disease and pathology were published [24,25]. Indeed, some miRNA function as viral host factors for diseases such as hepatitis C and Karposi’s sarcoma, are associated with cardiac and pulmonary disease, and are implicated in all stages of cancer, from initiation to tumor promotion and progression [26,27,28]. Investigations into miRNA and human disease have since taken a three-pronged approach:

(1) investigation of the contributions of miRNA regulation/dysregulation in disease pathology;
(2) the use of chemical modifiers to antagonize or restore key miRNA related to disease pathology;
(3) the identification of miRNA as serum biomarkers for disease diagnosis and prognosis.

However, while much emphasis has been placed on investigating the pathologic roles of miRNA, there is also significant interest in understanding the physiologic regulatory capacity of miRNA. Because miRNA have been suggested to regulate as much as 60% of human gene expression, these small RNA are thought to participate in and play an important role in the majority of biologic processes within the cell. Indeed, miRNA are involved in regulating cell timing, cell growth, cell death, and of interest in this review, cellular metabolism [29,30]. For instance, the liver-specific miR-122 has been implicated in the maintenance of hepatocyte development, but also impacts hepatic cholesterol and lipid metabolism [31]. Inhibition of miR-122 in mice and non-human primates contributes to reduced plasma cholesterol levels, decreased hepatic fatty acid synthesis, and repressed cholesterol synthesis [31,32]. Also, miR-33, which is encoded by an intron within sterol regulatory element-binding protein 2 (SREBP2), the dominant sterol regulatory element binding protein supporting cholesterol synthesis and uptake, acts in concert with its host gene (SREBP2) to control cholesterol homeostasis by regulating cholesterol efflux [33]. Furthermore, changes in miRNA expression have also been implicated in alterations in insulin sensitivity and glucose responsiveness, as well as in the control of mitochondrial metabolism and systemic energy homeostasis [34,35].

Although genetic approaches have provided essential information regarding miRNA function and biology, the effects of alterations in dietary intake and nutritional status on miRNA expression are only now beginning to be examined. Evidence continues to accumulate that suggests that nutrients can significantly impact miRNA expression and function [36]. While a majority of these findings were demonstrated using transformed cells or animal models wherein the expression of specific miRNA was knocked-down or over-expressed, the results remain quite compelling, and the study of the physiological miRNA response to dietary deficiencies will likely be the focus of many future investigations.

## 5. Regulation of Iron Metabolism

**Figure 2:**
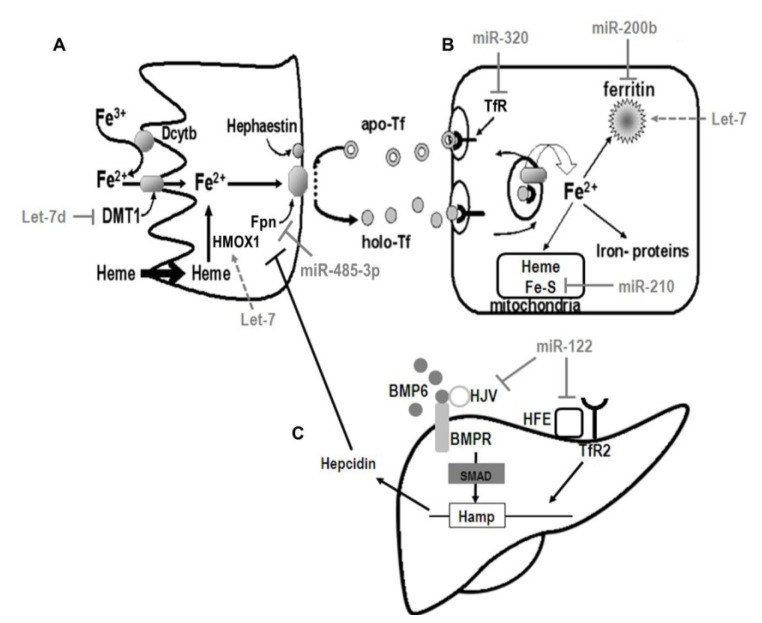
The potential roles for miRNA to influence iron metabolism. (**A**) Dietary iron absorption. Non-heme (Fe^3+^) iron may be reduced by duodenal cytochrome B (Dcytb) and transported into the cytosol by divalent metal transporter-1 (Dmt1). Dietary heme iron is transported across the apical membrane by mechanisms that remain unclear and iron is released from heme by heme oxygenase (Hmox1). Hmox1 expression is de-repressed via miR-let-7 targeting the transcriptional repressor Bach1. Iron that is neither stored nor utilized by the enterocyte is exported across the basolateral membrane by ferroportin-1 (Fpn) where it is oxidized by hephaestin before being bound to transferrin for transport to other tissues. Fpn-mediated iron export can be repressed through direct inhibition of Fpn by miR-485-3p. (**B**) Cellular iron uptake. The transferrin-bound iron binds to the transferrin receptor (TfR) on the plasma membrane. The Tf/TfR complex is internalized through receptor-mediated endocytosis and upon acidification of the endosome iron is released from Tf. The Tf/TfR complex can then be recycled back to the plasma membrane where the complex dissociates at a neutral pH. MiR-320 contributes to the regulation of cellular iron uptake by repressing TfR translation to decrease transferrin-dependent iron uptake. Endosomal iron released from Tf is carried into the cytoplasm by Dmt1, the expression of which may be repressed by miR-let-7d. The iron is then either stored in ferritin or utilized for iron-containing proteins. The regulatory action of miR-let-7 on Bach1 to de-repress ferritin transcription potentially enhances cytosolic iron storage. Utilization of iron is influenced directly by miR-210 which targets the Fe–S cluster assembly proteins Iscu1/2 thereby decreasing mitochondrial metabolism. (**C**) Systemic control of iron homeostasis. In the liver, Tf interacts with TfR2 and the protein Hfe to trigger the bone morphogenetic protein (BMP) and SMAD signaling cascade via interaction with the BMP co-receptor hemojuvelin (Hjv) to activate Hamp (hepcidin) transcription. The liver specific miR-122 directly targets HFE and HJV to contribute to the regulation of systemic iron homeostasis by decreasing hepcidin mRNA expression.

Many excellent reviews are available describing the molecular mechanisms coordinating mammalian iron homeostasis through the control of iron uptake, storage, and utilization [37,38,39]. Although a complete description will not be provided here, a brief overview of key regulators potentially affected by miRNA regulation is shown in Figure 2. Additionally, miRNA with validated roles in iron metabolism are summarized in Table 1.

### 5.1. Control of Systemic Iron Homeostasis

In the absence of a mechanism to promote iron efflux from the body, systemic iron homeostasis is tightly maintained through the regulation of absorption of iron from the intestine and recycling of iron from cells of the reticuloendothelial system (RES). Hepcidin is a key iron regulatory peptide hormone primarily responsible for coordinating systemic iron homeostasis by inversely affecting the rate of intestinal absorption and/or iron release from RES cells based on body iron stores [40]. When iron stores are elevated, hepcidin expression, synthesis, and secretion is increased to regulate systemic iron metabolism. Hepcidin acts to repress cellular iron export by binding to ferroportin (Fpn) and promoting its internalization, ubiquitination, and subsequent degradation [40,41]. Thus, iron is retained in the intestinal epithelium and the iron-recycling macrophages of the RES thereby decreasing serum iron levels. Conversely, when iron stores are low, hepcidin expression is suppressed and intestinal iron absorption and iron release from the RES cells is enhanced in an effort to restore iron homeostasis [41].

**Table 1:** Direct targets of miRNA associated with mammalian iron metabolism.

miRNA	Target mRNA	Reference(s)
miR-Let-7d	DMT (∆IRE), BACH1	Andolfo *et al.* (2010) [42], Hou *et al.* (2012) [43]
miR-122	HFE, HJV	Castoldi *et al.* (2009) [44]
miR-196	BACH1	Hou *et al.* (2010) [45]
miR-200b	FTH	Shpyleva *et al.* (2009) [46]
miR-210	ISCU, TFR	Chan *et al.* (2009) [47], Yoshioka *et al.* (2012) [48]
miR-214	Lactoferrin	Liao *et al.* (2010) [49]
miR-320	TFR	Schaar *et al.* (2009) [50]
miR-485-3p	FPN	Sangokoya *et al.* (2013) [51]
miR-584	Lactoferrin Receptor	Liao *et al.* (2010) [52]

### 5.2. Regulation of Cellular Iron Metabolism

Whereas hepcidin is considered to be the primary means of regulating systemic iron homeostasis, a family of cytosolic RNA binding proteins known as Iron Regulatory Proteins (IRP) is considered to be the global regulators of cellular iron homeostasis. IRP regulate cellular iron homeostasis by “sensing” intracellular iron status and accordingly coordinating iron uptake, storage, and utilization. The two members of this IRP family include IRP1 and IRP2, both of which are cytosolic RNA binding proteins that coordinate cellular iron homeostasis through high-affinity binding to Iron Responsive Elements (IRE) in either the 5′ or 3′UTR of mRNA encoding proteins involved in iron metabolism. IRP binding to IRE in either the 5′ or 3′UTR results in altered protein translation or mRNA stability, respectively [53].

IRP1 is a bifunctional protein that exhibits either high-affinity RNA binding activity (IRP1) or enzymatic activity by functioning as the cytosolic isoform of the TCA cycle enzyme mitochondrial aconitase (m-acon) [54]. Under iron-deficient conditions IRP1 is converted to its active RNA binding form and binds to IRE with high affinity, whereas under iron replete conditions the protein possesses enzymatic activity through the assembly of a [4Fe–4S] cluster to function as the cytosolic isoform of aconitase (c-acon) [54]. This so-called “Fe–S cluster switch” is the generally considered the primary means through which IRP1/c-acon activity is regulated, though other iron-independent mechanisms have been described and are reviewed elsewhere [37].

In contrast to IRP1, IRP2 does not contain a [4Fe–4S] cluster, functions only as an RNA binding protein, and is primarily regulated through modulation of protein stability and degradation via an iron-dependent polyubiquitination mechanism [55,56]. Under iron-replete conditions, IRP2 is targeted for proteasomal degradation by interacting with an E3 ubiquitin ligase complex containing F-box and leucine-rich repeat protein 5 (FBXL5) [57,58]. In the presence of iron, the hemerythrin domain of FBXL5 binds iron resulting in enhanced stability of the protein thereby promoting ubiquitination of IRP2. Under iron-deficient conditions, FBXL5 is destabilized and IRP2 protein accumulation is favored [57,58].

Fe–S clusters in proteins, such as IRP1, act as cofactors that are essential for numerous biologic processes including maintenance of iron homeostasis, mitochondrial respiration, electron transfer, metabolism, and many other regulatory functions [59]. Proteins containing Fe–S clusters are found in virtually all organisms, and within multiple cellular compartments including the mitochondria, cytosol, and nucleus. The synthesis and assembly of Fe–S clusters is a complex and highly regulated process involving the delivery of iron and sulfide to specific apoproteins located within specific subcellular compartments [59]. Mutations in genes involved in Fe–S cluster biogenesis, such as the Fe–S assembly proteins Iscu1/2, have been attributed to a spectrum of human diseases associated with a dysregulation in cellular iron metabolism [60,61]. Given the importance of Fe–S cluster-containing proteins in the regulation of iron homeostasis (*i.e.*, IRP1) and energy production (*i.e.*, m-acon), it is of interest to identify and elucidate regulatory factors involved in the formation and maintenance of Fe–S clusters, particularly in response to ID.

## 6. The Molecular Coordination of Iron Homeostasis by miRNA

In addition to understanding the potential impact of iron status on miRNA processing, it is of interest to determine the extent to which miRNA contribute to the regulation of iron metabolism. Since their discovery in 1993, miRNA have been identified as the largest subclass of non-coding RNA and are predicted to regulate anywhere from 30% to as much as 60% of all protein-coding genes [30]. Provided the increasing roles for miRNA to fine-tune gene expression and coordinate cellular functions, it is reasonable to speculate that nutrient availability or nutritional status might affect miRNA expression in an effort to maintain nutrient homeostasis. Thus characterizing factors that contribute to alterations in miRNA biogenesis, processing, and function will enhance our understanding of mechanisms by which cells respond to alterations to various situations such as changes in environmental conditions and nutrient (e.g*.*, iron) availability.

To date, an investigation into the degree to which *dietary* iron influences miRNA expression or regulation in humans has not been fully described. Despite the paucity of data in terms of altered miRNA expression in response to dietary intake, there is ample evidence indicating a potential role for miRNA to regulate both systemic and cellular iron homeostasis at multiple points by influencing iron absorption, transport, storage, and utilization (Figure 2). For instance, iron absorption and utilization may be affected by repression of the non-IRE (∆IRE) isoform of DMT1 by miR-let-7d [42]. Overexpression of miR-let-7d in K562 erythroleukemia cells suppressed expression of both DMT1 (∆IRE) mRNA and protein levels thereby decreasing the export of endosomal iron for use by the cell [42]. The decrease in endosomal iron export elicited an iron-deficient response, as evidenced by an increase in TfR expression, decreased ferritin protein abundance, and decreased hemoglobin content of the cell [42].

Iron acquisition is also likely subject to miRNA-dependent regulation. For example, overexpression of miR-210 decreases TfR protein abundance in MCF7 cells [48]. Furthermore, enhanced expression of miR-320 decreases the abundance of TfR on the plasma membrane and limits iron uptake in the lung carcinoma cell line A549 [50]. The multi-functional iron-binding protein lactoferrin, along with its receptor, is also regulated by miRNA in human cancer cells. Lactoferrin has been characterized as a functional target of miR-214 in both HC11 and MCF7 cells [49]. Interestingly the seed region aligning to miR-214 in the 3′UTR of lactoferrin is very highly conserved and identical in the lactoferrin 3′UTR of mouse, rat, pig, goat, camel, bovine, and human species [49]. The post-transcriptional expression of the lactoferrin receptor is mediated by miR-584 in both Caco-2 cells and in mouse small intestine during the perinatal period [52]. Cellular export may also represent a miRNA-mediated point of regulation in the maintenance of iron homeostasis as the mRNA encoding the only known cellular iron exporter FPN was recently shown to be targeted by miR-485-3p [51]. While the over-expression of miR-485-3p was associated with increased cellular iron levels, the inhibition of miR-485-3p expression decreased cellular iron content [51]. These findings provide evidence that miR-485-3p contributes to the coordination of iron homeostasis by regulating iron release through Fpn. In the absence of a regulated excretory pathway to rid the body of excess iron, the regulation of iron uptake or acquisition is a key point of control in maintaining cellular and systemic iron homeostasis. These exciting findings highlight the potential for miRNA to provide an additional means of control to fine-tune the regulation of cellular iron uptake.

In addition to the regulation of iron uptake and acquisition, miRNA may also contribute to the control of cellular iron homeostasis through regulation of iron storage via ferritin. The expression of both forms of the iron storage protein, heavy- or heart ferritin (FtH) and light- or liver ferritin (FtL) are significantly higher in human breast cancer cells with a particularly aggressive phenotype and correlates with a decreased expression of miR-200b [46]. The de-repression of FtH expression may be, at least in part, due to the presence of a miR-200b seed sequence in FtH [46]. Curiously, miR-200b has also been shown to correlate with dietary zinc depletion and repletion [62]. The functional and physiologic causes and consequences of miR-200b regulation in response to alterations in iron and zinc status will likely be the focus of future studies. Iron storage may also be indirectly affected by miRNA as both miR-196 and miR-let-7d target the heme-regulated transcriptional repressor Bach1 resulting in a de-repression of Bach1 targets such as HMOX1 and ferritin [43,45]. Although ferritin transcription may be reduced via Bach1, the capacity for miR-let-7d-dependent repression of Bach1 to de-repress ferritin expression and synthesis remains unknown [63].

Systemic iron homeostasis is also likely influenced by miRNA expression via the liver-specific miR-122 [44]. Inhibition of miR-122 by locked nucleic acid (LNA) modification is associated with an increased expression of hemochromatosis gene (HFE), hemojuvelin (HJV or HFE2), bone morphogenetic protein receptor type 1a (BMPR1A), and hepcidin (HAMP) mRNA, all of which contribute to a reduction in both plasma and liver iron, in addition to mildly impairing hematopoiesis [44]. In fact, both HFE and HJV are directly targeted by miR-122, suggesting that miR-122 could be targeted for therapeutic intervention for diseases of iron metabolism [44]. Intriguingly, miR-122 also correlates with copper accumulation and the onset of fulminant hepatitis in a rodent model of Wilson’s disease [64]. Elevated serum levels of miR-122 are detectable as much as two weeks earlier than traditional hepatitis-associated serum markers and therefore may represent a potential non-invasive biomarker for early detection of liver disease [64]. While it is tempting to postulate that miR-122 may be yet another interesting link between iron and copper metabolism, it is important to note that miR-122 comprises ~70% of all hepatic miRNA expression, and is therefore likely to have numerous regulatory roles and capacities [31,65].

Erythropoietic demands for iron to support the synthesis of hemoglobin remains a major factor in coordinating iron absorption and utilization, thus miRNA-dependent control of erythropoiesis has the potential to also contribute to the control of systemic iron homeostasis. Interestingly, many miRNA are highly expressed in the initial stages of erythropoiesis and a decline in their expression appears to be required for normal erythrocyte proliferation (miR-223), differentiation (miR-150), and maturation (miR-221/222) [66]. Conversely, miR-96 is actually more abundant in adult reticulocytes than umbilical cord blood, and contributes to the regulation of adult erythropoiesis via its direct interaction and repression of γ-globin [67]. The therapeutic potential for the manipulation of erythropoiesis via targeting of miRNA is the focus of considerable investigation.

Though the miRNA-dependent regulation of Fe–S cluster biogenesis and the potential effects on cellular iron metabolism via regulation IRP1 has been suggested, the effects of dietary iron intake or iron status on miRNA expression and Fe–S cluster assembly have not been extensively investigated. Current evidence suggests that the hypoxia-inducible miR-210 targets Fe–S cluster biogenesis and assembly via the regulation of the iron-sulfur cluster scaffold proteins Iscu1/2 [47,48]. Given the overlap between iron and/or oxygen sensing and maintenance of iron homeostasis, the potential for miR-210 to repress Fe–S cluster biogenesis, and thereby contribute to the regulation of IRP1 activity, remains of considerable interest. The effect of miR-210-dependent repression of Iscu1/2 expression on cellular metabolism is discussed below, though the role of miR-210 on IRP1 function (e.g*.*, RNA binding activity *versus* aconitase activity) and its impact on cellular iron homeostasis is not yet fully characterized.

## 7. microRNA Mediate the Reciprocal Relationship between Iron and Oxygen Homeostasis

Despite the cellular requirements for iron, it is important to note there is also potential for iron toxicity. In excess, the redox activity of “free” iron can lead to the production of damaging reactive oxygen species (ROS) and enhanced levels of oxidative stress [68]. The expression of the gene encoding the iron-containing enzyme heme oxygenase 1 (Hmox1) is induced by oxidative stress and can function as a key cytoprotective enzyme. HMOX1 gene expression is typically repressed by Bach1, but introduction of factors such as ROS or heme into the cell inhibits the DNA binding activity of Bach1 resulting in a de-repression of its target genes (e.g., HMOX1) [43]. Induction of the miR-let-7 miRNA also enhances HMOX1 expression by suppressing the regulatory capacity of Bach1 [43,45]. Excitingly, the indirect regulation of HMOX1 via miR-let-7 is sufficient to attenuate oxidant injury in human hepatocytes, and may represent a novel therapeutic approach for protecting cell integrity under conditions that induce oxidative stress, such as iron overload disorders [43].

In contrast to excess iron accumulation, when sufficient iron is unavailable hemoglobin production falls and the oxygen carrying capacity of the blood is diminished resulting in limited tissue oxygen delivery. Further complicating the relationship between oxygen and iron is the utilization of iron stores to maintain oxygen homeostasis in the presence of limited iron availability. When oxygen levels are low, iron stores are mobilized to enhance hemoglobin synthesis and erythrocyte production in an effort to restore tissue oxygen availability [69,70]. Therefore, under conditions of ID when there are insufficient iron stores to meet erythropoietic demands, it is reasonable to hypothesize that molecular mechanisms would exist to coordinate iron availability with iron utilization in a low oxygen environment. The discovery of an IRE in the 5′UTR of the oxygen-sensitive transcription factor hypoxia inducible factor-2α (HIF-2α) was an exciting finding that confirmed the potential for cross-talk between iron and oxygen sensors [71]. Under ID conditions when IRP is active, the translation of HIF-2α is repressed which serves to attenuate the induction of HIF-2α target genes [72]. In fact, in animals lacking IRP1, there is a profound increase in iron absorption and erythropoiesis due to a de-repression of HIF-2α-mediated signaling [73].

The cellular adaptation to hypoxia also requires an adjustment in iron utilization to appropriately reduce cellular metabolism and oxygen consumption. Fe–S cluster-containing enzymes are critical to the function of the ETC and TCA cycle, and a reduction in the activity of these enzymes can result in a reduction in cellular respiration and energy production. The oxygen-sensitive transcription factor HIF-1α plays a key regulatory role by coordinating oxygen availability with cellular metabolism by increasing glycolytic gene expression and promoting glucose uptake and utilization [74]. Interestingly, a hypoxia response element-binding site for HIF-1α was identified in the promoter of miR-210, a miRNA demonstrated to repress the Fe–S cluster assembly proteins Iscu1/2 [47,75]. This relationship between HIF-1α and miR-210 extends the role through which HIF-1α coordinates cellular metabolism with oxygen availability. In fact, the miR-210-dependent repression of Iscu1/2 is associated with a decline in mitochondrial energy production [47]. In human breast cancer cells, the miR-210-mediated repression of Iscu1/2 expression is also linked to the regulation of iron homeostasis via the modulation of Fe–S cluster assembly in IRP1 and through a miR-210 seed sequence in the 3′UTR of TfR that would, perhaps paradoxically, limit Tf-dependent iron uptake [48]. The importance of miRNA similar to miR-210 and miR-320 in the regulation of iron metabolism *in vivo* and in non-cancerous cell lines remains to be established, though these findings may represent potential therapeutic targets for sequestering iron from cancerous and tumorigenic cell types.

## 8. Future Developments in the Role of miRNA in the Regulation of Iron Homeostasis

Though much of our understanding of the effects of miRNA on the regulation of nutrient homeostasis and metabolism have been derived from genetic approaches (e.g*.*, miRNA knock-out models, transgenic over-expression of miRNA, or through the use of specific inhibitors), the study of miRNA under physiologic conditions is considerably more complicated for a multitude of reasons. First, by the nature of their mode of regulation, it is difficult to detect modest but significant changes in miRNA expression associated with alterations in nutrient status as a result of dietary intake. The relatively modest changes in miRNA expression may make it difficult to discern physiological significance at the level of the entire organism. Furthermore, because miRNA generally function in a homeorhetic manner to “fine-tune” gene expression (and protein translation), alterations in target mRNA expression are sometimes difficult to assess, especially in response to changes in dietary intake or nutrient availability. Other important factors to be considered when examining the functionality of miRNA *in vivo* include developmental timing, level of expression (of both miRNA and target mRNA), and methods used to assess the regulation of putative or validated target mRNA (e.g., measuring changes at the mRNA or protein level).

Additionally, based on current predictions, the targets of the majority of miRNA have yet to be identified and experimentally validated. While current algorithms designed for predicting miRNA:mRNA interactions *in silico* are becoming progressively more sophisticated, the validation of these targets remains an arduous task as most individual miRNA species are thought to have hundreds of potential target mRNA. Lastly, it is also important to question which form of miRNA (*i.e.*, pri-, pre-, or mature miRNA) is most appropriate for biological assessment. In terms of studying the impact of alterations in iron status on miRNA expression, choosing which form of a miRNA to examine is particularly important, especially since iron status may impact miRNA processing.

Despite the inherent challenges associated with interrogating the impact of nutrient status on miRNA expression and regulation, the pursuit of identifying these relationships between nutrient status (e.g., iron deficiency) and miRNA expression is warranted as the molecular mechanisms coordinating miRNA regulation and iron homeostasis are not yet fully understood or characterized. Furthermore, as demonstrated in the field of cancer research, many possibilities exist for exploiting both the therapeutic potential of miRNA for the treatment of diseases of iron metabolism and for the identification of plasma miRNA biomarkers that might be sensitive and timely indicators of changes in an individual’s iron status. For example, the repression of DMT1 by the overexpression of miR-let-7d might represent a potential mechanism protecting against cellular damage and neurodegeneration under conditions of iron excess. Conversely, antagonizing the expression of miR-96 may provide a therapeutic approach to the treatment of hemoglobinopathies by allowing for the increased expression of fetal hemolgobin (α_2_γ_2_) to compensate for a reduced or abnormal adult hemoglobin (α_2_β_2_) expression. Finally, it remains to be established whether many of the miRNA demonstrated to affect iron metabolism using cell-based or other genetic approaches, such as miR-320 and miR-200b, have physiological roles *in vivo* or in non-transformed cell types, especially in response to physiologically-relevant alterations in nutrient intake. The potential for miRNA to serve as therapeutic targets for the treatment of genetic disorders or in neoplastic disease is quite exciting, though given the pleiotropic effects of altering miRNA function, it is important that we possess a more thorough understanding of miRNA biology and function. In light of the progress that has been made over the last decade regarding miRNA expression, biogenesis, processing, and function, nutritional scientists are well-positioned to examine the relationship between nutrient (e.g., iron) status and miRNA and provide insight into mechanisms coordinating nutrient-gene interactions.

## 9. Conclusions

The ability to fine-tune gene expression by miRNA provides cells an additional level of control when adapting to changes in the cellular environment. In terms of allowing cells to adapt to changes in iron status, miRNA are involved in regulating cellular iron uptake, storage, and utilization. More specifically, miRNA post-transcriptionally regulate the expression of genes associated with iron acquisition (TFR and DMT1), iron export (FPN), iron storage (ferritin), iron utilization (ISCU), and coordination of systemic iron homeostasis (HFE and HJV). Given the diversity of miRNA expressed in cells among different tissues and number of potential mRNA targets, characterizing conditions (e.g., iron deficiency and iron overload) that contribute to alterations in miRNA expression will further enhance our understanding of mechanisms by which cells respond to changes in iron demand and/or iron availability to control cellular iron homeostasis.

## Abbreviations

Ago: Argonaute
BMP: bone morphogenetic protein
c-acon: cytosolic aconitase
Dcytb: duodenal cytochrome b
DGCR8: DiGeorge syndrome critical region 8
DMT1: divalent metal transporter 1
FBXL5: F-box and leucine-rich repeat protein5
Fe–S: iron-sulfur
Fpn: ferroportin
Hamp: hepcidin
HFE: hemochromatosis gene
HIF: hypoxia inducible factor
Hjv: hemojuvelin
Hmox1: heme oxygenase 1
IRE: iron responsive element
IRP: iron regulatory protein
Iscu: iron-sulfur cluster scaffold homolog
LNA: locked nucleic acid
m-acon: mitochondrial aconitase
miRNA: microRNA
Pcbp2: poly(rC) binding protein 2
pre-miRNA: precursor miRNA
pri-miRNA: primary miRNA
RES: reticuloendothial system
RISC: RNA-induced silencing complex
ROS: reactive oxygen species
Srebp2: sterol regulatory element binding protein 2
Tf: transferrin
TfR: transferrin receptor
UTR: untranslated region
